# Analysis of the Vaccine Potential of Plasmid DNA Encoding Nine Mycolactone Polyketide Synthase Domains in *Mycobacterium ulcerans* Infected Mice

**DOI:** 10.1371/journal.pntd.0002604

**Published:** 2014-01-02

**Authors:** Virginie Roupie, Sacha J. Pidot, Tobba Einarsdottir, Christophe Van Den Poel, Fabienne Jurion, Timothy P. Stinear, Kris Huygen

**Affiliations:** 1 Service Immunology, Scientific Institute of Public Health (WIV-ISP Site Ukkel), Brussels, Belgium; 2 Department of Microbiology and Immunology, University of Melbourne, Parkville, Victoria, Australia; Kwame Nkrumah University of Science and Technology (KNUST) School of Medical Sciences, Ghana

## Abstract

There is no effective vaccine against Buruli ulcer. In experimental footpad infection of C57BL/6 mice with *M. ulcerans*, a prime-boost vaccination protocol using plasmid DNA encoding mycolyltransferase Ag85A of *M. ulcerans* and a homologous protein boost has shown significant, albeit transient protection, comparable to the one induced by *M. bovis* BCG. The mycolactone toxin is an obvious candidate for a vaccine, but by virtue of its chemical structure, this toxin is not immunogenic in itself. However, antibodies against some of the polyketide synthase domains involved in mycolactone synthesis, were found in Buruli ulcer patients and healthy controls from the same endemic region, suggesting that these domains are indeed immunogenic. Here we have analyzed the vaccine potential of nine polyketide synthase domains using a DNA prime/protein boost strategy. C57BL/6 mice were vaccinated against the following domains: acyl carrier protein 1, 2, and 3, acyltransferase (acetate) 1 and 2, acyltransferase (propionate), enoylreductase, ketoreductase A, and ketosynthase load module. As positive controls, mice were vaccinated with DNA encoding Ag85A or with *M. bovis* BCG. Strongest antigen specific antibodies could be detected in response to acyltransferase (propionate) and enoylreductase. Antigen-specific Th1 type cytokine responses (IL-2 or IFN-γ) were induced by vaccination against all antigens, and were strongest against acyltransferase (propionate). Finally, vaccination against acyltransferase (propionate) and enoylreductase conferred some protection against challenge with virulent *M. ulcerans* 1615. However, protection was weaker than the one conferred by vaccination with Ag85A or *M. bovis* BCG. Combinations of these polyketide synthase domains with the vaccine targeting Ag85A, of which the latter is involved in the integrity of the cell wall of the pathogen, and/or with live attenuated *M. bovis* BCG or mycolactone negative *M. ulcerans* may eventually lead to the development of an efficacious BU vaccine.

## Author Summary

Buruli ulcer (BU) is an infectious disease, characterized by deep, ulcerating skin lesions, particularly on arms and legs, which are provoked by a toxin. BU is caused by a microbe of the genus that also cause tuberculosis and leprosy. The 33 countries where Buruli ulcer has been detected, especially in West Africa, have mainly tropical and subtropical climates, although the disease is also present in temperate areas of Australia and Japan. There is no effective vaccine against BU and it is still not fully understood which immune defence mechanisms (antibodies and/or T cells) are needed to control the infection. The identification of microbial components that are involved in immune control is an essential step in the development of an effective vaccine. In this paper, we used an experimental mouse model to demonstrate the immunogenicity and the vaccine potential of enzymes involved in the toxin synthesis. Combinations with other vaccine candidates, such as a subunit vaccine against Ag85A targeting cell wall synthesis or with live, attenuated *M. bovis* BCG or mycolactone negative *Mycobacterium ulcerans* remain to be tested.

## Introduction

Buruli ulcer (BU) is a necrotizing bacterial skin disease caused by *Mycobacterium ulcerans*. *M. ulcerans* produces a diffusible macrolide toxin, called mycolactone (ML) which is essential for bacterial virulence [1]. BU has been documented in 33 countries worldwide, although most of the cases occur in West Africa, primarily Benin, Côte d'Ivoire, Ghana and more recently Gabon. According to the World Health Organization, about 5000 cases annually are reported from 15/33 countries. The incidence in endemic regions of Ghana has been estimated at 150 cases/100 000 inhabitants. However, as the disease is not notifiable in many countries and most patients live in remote, rural areas with little medical infrastructure, the actual number of cases is likely to be much higher. Regardless, as the disease burden is mostly localized to certain geographical areas, the impact of vaccination and treatment efforts can be very high [2].

Prevention of BU is complicated by the fact that while *M. ulcerans* is present in the environment in disease endemic areas [3], [4], the route of transmission is largely unknown. In Australia, infection following contamination of a golf course irrigation system was reported [5] while many cases elsewhere are related to disruption of the environment, e.g. due to deforestation and building of dams [4]. Possible sources of infection include aquatic insects, mosquitoes and mammals [6], [7]. In temperate south-eastern Australia (State of Victoria) ringtail and brushtail opposums presenting typical ulcerative lesions have been identified and *M. ulcerans* DNA was detected at high level by real-time qPCR in faeces of these animals [8]. Person-to-person transmission appears to be extremely rare [9].

*M. ulcerans* is distinct from other mycobacteria in that it produces a lipid toxin (ML), which is synthesized by three large polyketide synthases encoded by *mlsA1*, *mlsA2* and *mlsB* localized on the 174 kb pMUM001 virulence plasmid [10]. These synthases are composed of different modules, which each have a particular sequence of enzymatic domains. ML locally suppresses T cell responses at non-toxic levels [11]. This T cell suppression induced by ML is not completely understood, but it is clear that ML can alter both early signaling at the T cell receptor level by activation of the Src-family kinase Lck as well as blocking cytokine responses at a post-transcriptional level [11]. At higher concentrations, the toxin is cytotoxic Using a semiquantitative reverse transcription-PCR analysis of mRNA isolated from BU lesions, we have shown that production of IL-10 rather than production of IL-4 or IL-13 by Th2-type T cells may be involved in the low *M. ulcerans*-specific IFN-gamma response in Buruli disease patients [12]. A more in depth study by R. Phillips *et al* on serum from 37 BU patients from Ahafo Ano North District of Ghana demonstrated by use of Luminex technology that patients with active ulcers display a distinctive profile of immune suppression, marked by the down-modulation of four inflammatory chemokines: macrophage inflammatory protein (MIP) 1β, IL-8, monocyte chemoattractant protein (MCP) 1, and (to a lesser extent) fractalkine [13]. These immunological defects were induced early in the disease and resolved after anti-BU therapy [13]. An impaired capacity to produce Th1, Th2, and Th17 cytokines on stimulation with the mitogen Phytohaemagglutinin PHA was also observed in the Phillips' study (be it on a limited number of 4 patients with BUD and 4 healthy control participants) [13]. Interestingly, some of the defects in cytokine and chemokine response could be mimicked *in vitro* by incubation of CD4+ peripheral blood lymphocytes with ML [13].

ML is an obvious candidate for a BU vaccine, but by virtue of its chemical composition and possibly because of its immunosuppressive properties, the toxin is not immunogenic and in neither infected mice nor humans ML-specific antibodies have been found. However, antibodies against some of the polyketide synthase domains involved in ML synthesis, were found in BU patients and healthy controls from the same endemic region, suggesting that these domains are indeed immunogenic [14].

Aiming to interfere with ML synthesis, we have used a DNA prime/protein boost strategy targeting nine of these polyketide synthase domains. C57BL/6 mice were vaccinated against three variations of the acyl carrier protein domain (ACP1, ACP2, ACP3), against three acyltransferase domains (ATac1, ATac2, and ATp), against the enoylreductase domain (ER), against one of the ketoreductase domains (KR A) and against the ketosynthase load module domain (KS).

## Methods

### Mice

C57BL/6 mice were bred in the Animal Facilities of the WIV-ISP (Site Ukkel), from breeding couples originally obtained from JANVIER SAS in Le Genest Saint Isle, France. Mice were 8–10 weeks old at the start of the experiments. Female mice were used for immune analysis and male mice for the protection studies.

### Mycobacterial strains and antigens

Virulent *M. ulcerans* 1615 strain (Malaysia) [10] was kindly given to us by Dr. P. Small (University of Tennessee). Bacteria were maintained and amplified *in vivo* in mouse footpad [15]. *M. bovis* BCG strain GL2 was grown for 2 weeks as a surface pellicle at 37°C on synthetic Sauton medium and homogenized by ball mill as described before and kept at −80°C in 20% of glycerol until used [16].

### Recombinant proteins

Bacterial expression vector pET-DEST42 encoding the genes of 8 enzymatic modules, ACP1, ACP2, ACP3, ATac1, ATac2, ATp, ER and KS or pDEST17 vector encoding KR A (all as C-terminally Histidine-tagged proteins), were constructed at the University of Melbourne, Australia and used for transformation and selection in *E. coli* BL-21. Following induction with IPTG for 2–4 hours, cells were lysed and recombinant proteins were purified according to standard protocol on immobilized metal affinity chromatography (IMAC) using gravity flow. Recombinant Ag85A protein from *M. ulcerans* (MUL 4987) was kindly given to us by Dr. G. Pluschke (Swiss Tropical and Public Health Institute, Basel, Switzerland).

**Figure 1:**
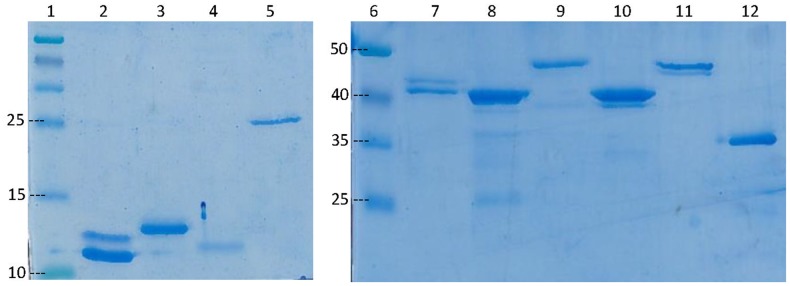
IMAC purified polyketide synthase domains and Ag85A (MUL4987) separated by SDS-PAGE. Recombinant proteins (2,5 µg each) were separated by 15% (left) or 12% (right) SDS-PAGE and stained with PageBlue™. Lane 1, molecular weight markers (kD); 2, ACP1; 3, ACP2; 4, ACP3; 5, KR A; 6, molecular weight markers (kD); 7, ATac1; 8, Atac2; 9,ATp; 10, ER; 11, KS; 12, Ag85A (MUL4987).

**Figure 1** shows the IMAC purified Pks domains and MUL4987 separated in 15% (left figure) or 12.5% SDS-PAGE (right figure) and stained with Protein Staining Solution (Thermo Scientific, Rockford, Illinois, USA) .

### Plasmid DNA constructions

The genes encoding the nine enzymatic modules of the polyketide synthases were cloned in the eucaryotic expression vector pV1.Jns-tPA [17]. In this plasmid, the genes are expressed under the control of the promoter of IE1 antigen from cytomegalovirus, including intron A, preceded by the signal sequence of human tissue plasminogen activator

Briefly, sequences were amplified by PCR (Expand High Fidelity PCR System, Roche), from the corresponding pET-DEST42 and pDEST17 constructs. Primers used for cloning are shown in Table 1.

**Table 1:** Primers used for the cloning of nine Pks domains.

Gene	Abbreviation	Primers for cloning in pV1J.ns-tPA	Enzyme used
Acyl carrier protein type 1	ACP-1	5′-aat**AGATCT**tgcacacccccgaaa-3′ 5′-att**AGATCT**tcatgtggggtgatcg-3′	*Bgl* II
Acyl carrier protein type 2	ACP-2	5′-aat**AGATCT**tggatcaagccgcatc-3′ 5′-gcc**AGATCT**tcagggtgtg-3′	*Bgl* II
Acyl carrier protein type 3	ACP-3	5′-aat**AGATCT**tgcacacccccgaaa-3′ 5′-att**AGATCT**tcaggtgtgcaggtgttg-3′	*Bgl* II
Type 1 acyltransferase with acetate specificity	ATac-1	5′-tac**TGATCA**tgcgtctgtaccagcatctc-3′ 5′-gta**TGATCAT**caatagtcaggtgaggcgagtt-3′	*Bcl* I
Type 2 acyltransferase with acetate specificity	ATac-2	5′-tat**AGATCT**tgtttgtgccctgggtgatttc-3′ 5′-tga**AGATCT**tcagtggtccacccagtaac-3′	*Bgl* II
Acyltransferase with propionate specificity	ATp	5′-atc**TGATCA**tggcagtgggtgtactgg-3′ 5′-gat**TGATCA**tcacattgtggtggtgtcgt-3′	*Bcl* I
Enoylreductase	ER	5′-tac**GGATCC**tgcttgacaccaccggcaa-3′ 5′-gat**GGATCC**tcacatgtcacgtaaggccg-3′	*BamH* I
Type A ketoreductase domain	KR A	5′-aat**AGATCT**tgagggggaccgtgt-3′ 5′-att**AGATCT**tcaggcgagcgaatc-3′	*Bgl* II
Load module ketosynthase domain	KS	5′-ggccggtg**AGATCT**tgaattt-3′ 5′-att**AGATCT**tcagccacccatggaa-3′	*Bgl* II

Primers used for the cloning of nine Pks domains in plasmid pV1J.ns-tPA. Restriction enzymes used are indicated. Sense primers are listed first and antisense primers second, within primer pairs. All restriction sites are shown in bold.

The amplified sequences were digested with *Bgl* II, *Bcl* I, *or BamH* I, purified on agarose (QIAkit PCR Purification kit, Qiagen) and T4 ligated into pV1.Jns-tPA vector digested with *Bgl* II. After ligation and transformation into DH5-α chemically competent *E. coli* cells (Invitrogen), clones were screened on LB-kanamycin medium (50 µg/mL) and plasmid was checked by restriction digestion and sequencing.

Plasmid DNA encoding the mature 32 kD Ag85A from *M.ulcerans* in V1J.ns-tPA vector was prepared as described before [18].

### Vaccination protocols

C57BL/6 were anesthesized by intraperitoneal injection of ketamine-xylazine and injected intramuscularly (i.m) in both quadriceps muscles with 2×50 µg plasmid V1-Jns-tPA encoding one of the nine polyketide synthase domains, empty vector as negative control and V1-Jns-tPA-Ag85A (MUL4987) as positive control on day 0 and day 21. On day 42, mice were injected subcutaneously (s.c.) in the back with 10 µg of corresponding, recombinant protein emulsified in Gerbu adjuvant, i.e. water miscible, lipid cationic biodegradable nanoparticles, completed with immunomodulators and GMDP glycopeptide (GERBU Biochemicals).

C57BL/6 mice were vaccinated intradermally with 1×10^5^ colony forming units (CFU) of *M. bovis* BCG strain GL2 on day 0.

### Cytokine production

Vaccinated mice were sacrificed 3 or 6 weeks after the third immunization. Spleens were removed aseptically and homogenized in a loosely fitting Dounce homogenizer and cells were adjusted to 4×10^6^ white blood cells/ml in RPMI-1640 medium (Gibco, Grand Island, NY) supplemented with 10% fetal calf serum (FCS), 5×10^−5^ M 2-mercapto-ethanol, penicillin, streptomycin and Polymyxin B sulphate (30 µg/ml, Sigma). Cells were cultivated at 37°C in a humidified CO_2_ incubator in round-bottom microwell plates individually and analyzed for Th1 type cytokine response to corresponding recombinant protein (5 µg/ml). Supernatants from at least three wells were pooled and stored frozen at −20°C. Cytokines were harvested after 24 h (IL-2) and 72 h (IFN-γ), when peak values of the respective cytokines can be measured.

### IL-2 ELISA

IL-2 activity was quantified by sandwich ELISA using coating antibody anti-mouse interleukine-2 (14-7022, eBioscience) and biotinylated detection antibody anti-mouse IL-2 (JES6-5H4, 13-7021, eBioscience). The detection limit of the IL-2 ELISA is 5 pg/ml.

### IFN- γ ELISA

IFN-γ activity was quantified by sandwich ELISA using coating antibody R4-6A2 and biotinylated detection antibody XMG1.2 (both BD Pharmingen). The detection limit of the IFN-γ ELISA is 5 pg/ml.

### IFN- γ ELISPOT

Antigen-specific spleen cell IFN-γ secretion was also assayed by ELISPOT as described earlier. Briefly, 96-well flat-bottomed nitrocellulose plates (MAHA S4510, Millipore, Billerica, MA) were incubated overnight at 4°C with 50 µl of capture purified anti-mouse IFN-γ (15 µg/ml; BD Pharmingen, Erembodegem, Belgium) in phosphate-buffered saline (PBS) and then saturated with 200 µl/well of RPMI-complete medium 2 h at 37°C. 180 µl of spleen lymphocytes (pool of four mice per group) were added at a cell concentration of 4.10^6^ cells/ml in the presence or absence of 20 µl proteins (5 µg/ml) and plates were incubated for 48 h at 37°C, 5% CO2. After extensive washing, plates were incubated 2 h at 37°C, 5% CO2 with 50 µl of biotinylated rat anti-mouse IFN-γ (2 µg/ml) (BD Pharmingen), washed and incubated for 45 min at 37°C, 5% CO2 with alkaline phosphatase labelled ExtrAvidine (Sigma-Aldrich, Bornem, Belgium). After washing, spots were revealed with Bio-Rad (Hercules, CA) alkaline phosphatase conjugate substrate kit, following the manufacturer's instructions and plates were analysed on a Bioreader-3000 LC (BioSys, Germany). Results are shown as mean spot-forming cells (SFC) per million lymphocytes.

### Antibody ELISA

Sera from C57BL/6 mice were collected by tail bleeding three and six weeks after the protein boost or six weeks after *M. ulcerans* challenge. Antigen-specific total immunoglobulin G (IgG) was determined by an enzyme-linked immunosorbent assay (ELISA) on serial dilutions of individual sera. The corresponding recombinant protein was used for coating (500 ng/well). Total antibody was detected using peroxidase-labeled rat anti-mouse immunoglobulin IgG (Experimental Immunology Unit, Université Catholique de Louvain, Brussels, Belgium) and orthophenylenediamine (Sigma) for revelation. Data are presented as the mean optical density at 490 nm (O.D_490 nm_) for 3–5 vaccinated mice tested individually for serum diluted 1∶50 and for 9 serial twofold dilutions thereof.

### *M. ulcerans* challenge

Six weeks after the protein boost (12 weeks after BCG), 15 mice/group were challenged with *M. ulcerans* 1615. 10^5^ acid fast bacilli (AFB) obtained by *in vivo* passage in footpad, were injected in the right footpad of the vaccinated mice. The number of bacilli injected, suspended in Dubos Broth Base medium (Difco), was determined by counting under a microscope after Ziehl-Neelsen staining. Viability of the *M. ulcerans* inoculum was checked by plating on 7H11 Middlebrook agar, supplemented with oleic-acid-albumin-dextrose-catalase enrichment medium. Yellow colonies were counted after 8 weeks of incubation at 32°C. The number CFU equaled the number of AFB.

### Analysis of protective efficacy

Five mice per group were sacrificed for enumeration of AFB six weeks after *M. ulcerans* challenge in the footpad. Briefly, the skin and bones were removed from infected footpad. Tissues were homogenized in a Dounce homogenizer and suspended in 2 ml of Dubos broth based medium containing glass beads. The number of AFB was counted on microscope slides after Ziehl-Neelsen staining.

Protection was also evaluated in ten mice/group by monitoring footpad swelling after *M. ulcerans* 1615 infection. The swelling was measured with a calibrated Oditest apparatus with a resolution of 0.01 mm as described previously [19]. Animals were euthanized when footpad swelling exceeded 4 mm according to the rules of the local ethical commission and survival curves were established.

### Statistical analysis

For cytokine production analysis, antibody production and AFB counting, statistical analysis was made according to one-way ANOVA test. Subsequent multiple comparisons between the different groups of animals and the antigens used was made by a Tukey's correction test. Statistical results are represented in the figure by *** (p<0.001), ** (p<0.01) and * (p<0.05). Median survival time was calculated using GraphPad, Log-rank (Mantel-Cox) test.

## Results

### Antigen specific IgG antibody responses in DNA primed-protein boosted C57BL/6 mice against nine Pks domains and Ag85A in *M. ulcerans* infected mice

**Figure 2:**
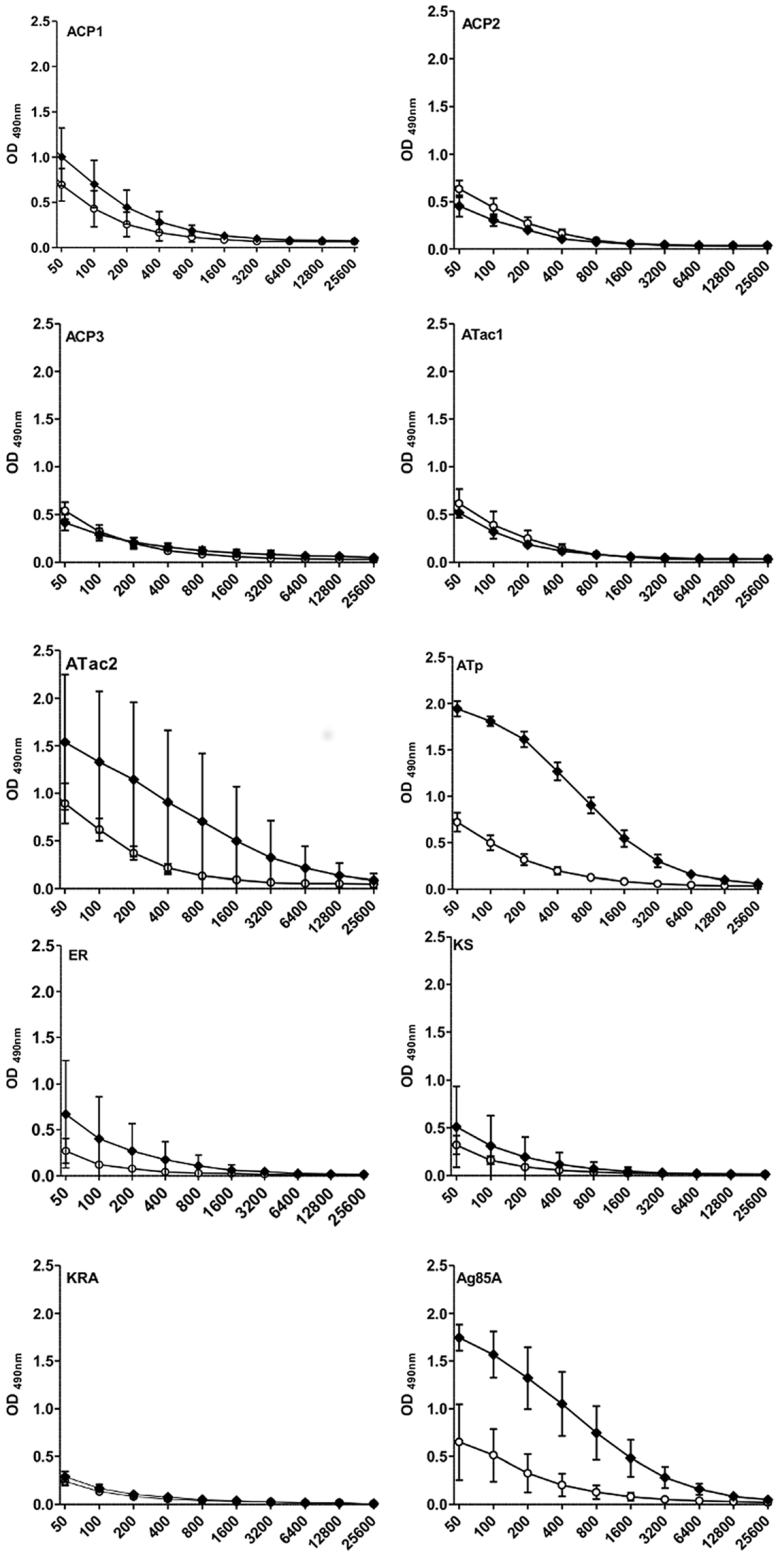
Antigen-specific IgG antibodies in naïve and vaccinated mice. IgG antibodies in C57BL/6 mice vaccinated twice with pDNA encoding the nine Pks synthase domains and boosted with the homologous recombinant protein. Sera were collected three weeks after the protein boost and tested by ELISA, using serial twofold dilutions, starting at 1∶50. Open circles: naive mice, closed circles vaccinated mice. Results presented as mean O.D. values ± SD of 4–6 mice tested individually.

As shown in **Figure 2**, vaccination against some of the Pks domains induced significant IgG antibodies. In particular, strong responses were found at three weeks after the protein boost in mice vaccinated against ATac2 and ATp. Vaccination against ACP1 and ER induced a weak IgG response (only 1/4 mice reactive), whereas IgG levels induced by vaccination against ACP2, ACP3, ATac1 KR A and KS were not different from IgG levels in naïve mice. Confirming previous findings [18], vaccination against Ag85A also induced strong antibody levels. At six weeks post vaccination (**Supplementary Figure S1**), IgG antibodies directed against ATac2 and ATp were still present, but lower than at week 3. ACP1 and Ag85A specific antibody levels remained at the same level, and ER specific antibodies were clearly higher at week 6 than at week 3 post protein boost (albeit with more variation between the 6 mice, antibody levels in 2/6 mice being lower than in the other 4 mice). IgG levels induced by vaccination against ACP2, ACP3, ATac1, KR A and KS remained negative.

### Antigen-specific spleen cell Th1 type cytokine secretion in C57BL/6 mice vaccinated against nine Pks domains and Ag85A using a DNA prime-protein boost protocol

Production of two Th1 type cytokines was analyzed in spleen cell culture supernatant of vaccinated mice stimulated with their respective antigens: Interleukin-2 and IFN-γ. IL-2 is a pleiotropic cytokine produced after antigen activation that plays pivotal roles in the immune response. Discovered as a T cell growth factor, IL-2 additionally promotes CD8+ T cell and natural killer cell cytolytic activity and modulates T cell differentiation programs in response to antigen, promoting naïve CD4+ T cell differentiation into T helper 1 (Th1) and T helper 2 (Th2) cells while inhibiting T helper 17 (Th17) and T follicular helper (Tfh) cell differentiation. Moreover, IL-2 is essential for the development and maintenance of T regulatory cells and for activation-induced cell death, thereby mediating tolerance and limiting inappropriate immune reactions [20]. The macrophage-activating cytokine IFN-γ on the other hand together with TNF-α is a well known pivotal cytokine in the control of mycobacterial infections, as illustrated by the increased susceptibility to tuberculosis in IFN-γ gene disrupted mice [21], [22]. Whereas IL-2 is produced exclusively by CD4+ T cells, IFN-γ can be produced by both CD4+ and CD8+ T cells, and therefore analysis of both cytokines may give complementary information.

**Figure 3:**
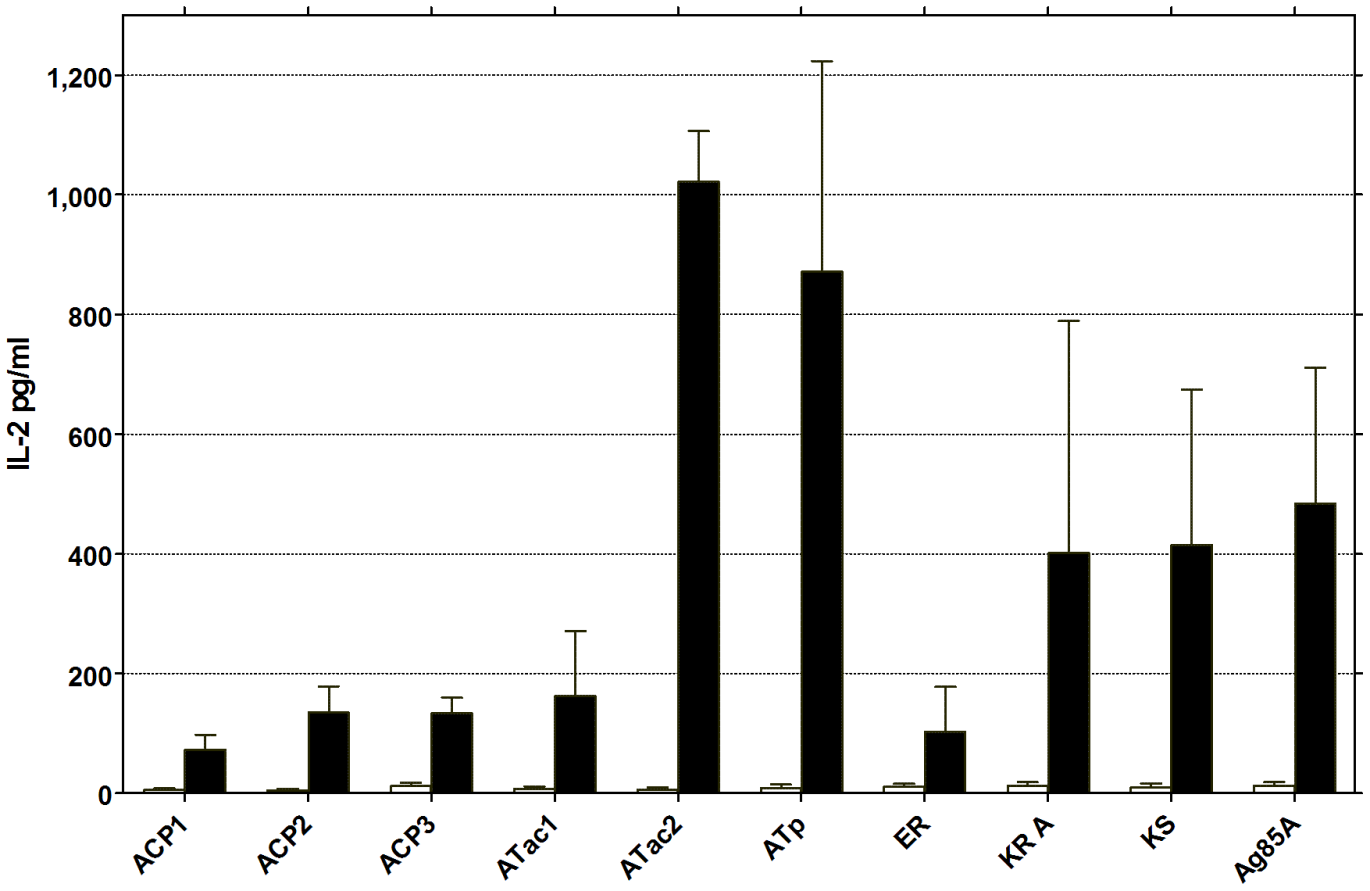
Antigen-specific IL-2 production in naïve and vaccinated mice, as tested by ELISA. Interleukin-2 levels in 24 hr spleen cell culture supernatants of C57BL/6 mice vaccinated against the 9 PkS domains using the pDNA prime/protein boost protocol and stimulated *in vitro* with the corresponding recombinant protein antigen (5 µg/ml). Results represent mean ± SD IL-2 values (pg/ml) of 4–6 mice tested individually. Data are representative of one of three experiments.

Vaccination against ATac2, ATp, KR A, KS and Ag85A resulted in significant spleen cell IL-2 production in 24 hr culture supernatant, ranging between 400 and 1000 pg/ml when cells were stimulated *in vitro* with the corresponding antigen (**Figure 3**). The same two Pks domains ATac2 and ATp, that induced strong antibodies, were also good inducers of IL-2. In contrast, vaccination against KR A (which did not induce an antibody response) also induced a good IL-2 response. Vaccination against ACP1, ACP2, ACP3, ATac1 and ER induced only very modest IL-2 levels between 100 and 200 pg/ml. Stimulation of cells from unvaccinated mice with the recombinant proteins induced IL-2 levels were close to the detection limit (5 pg/ml).

**Figure 4:**
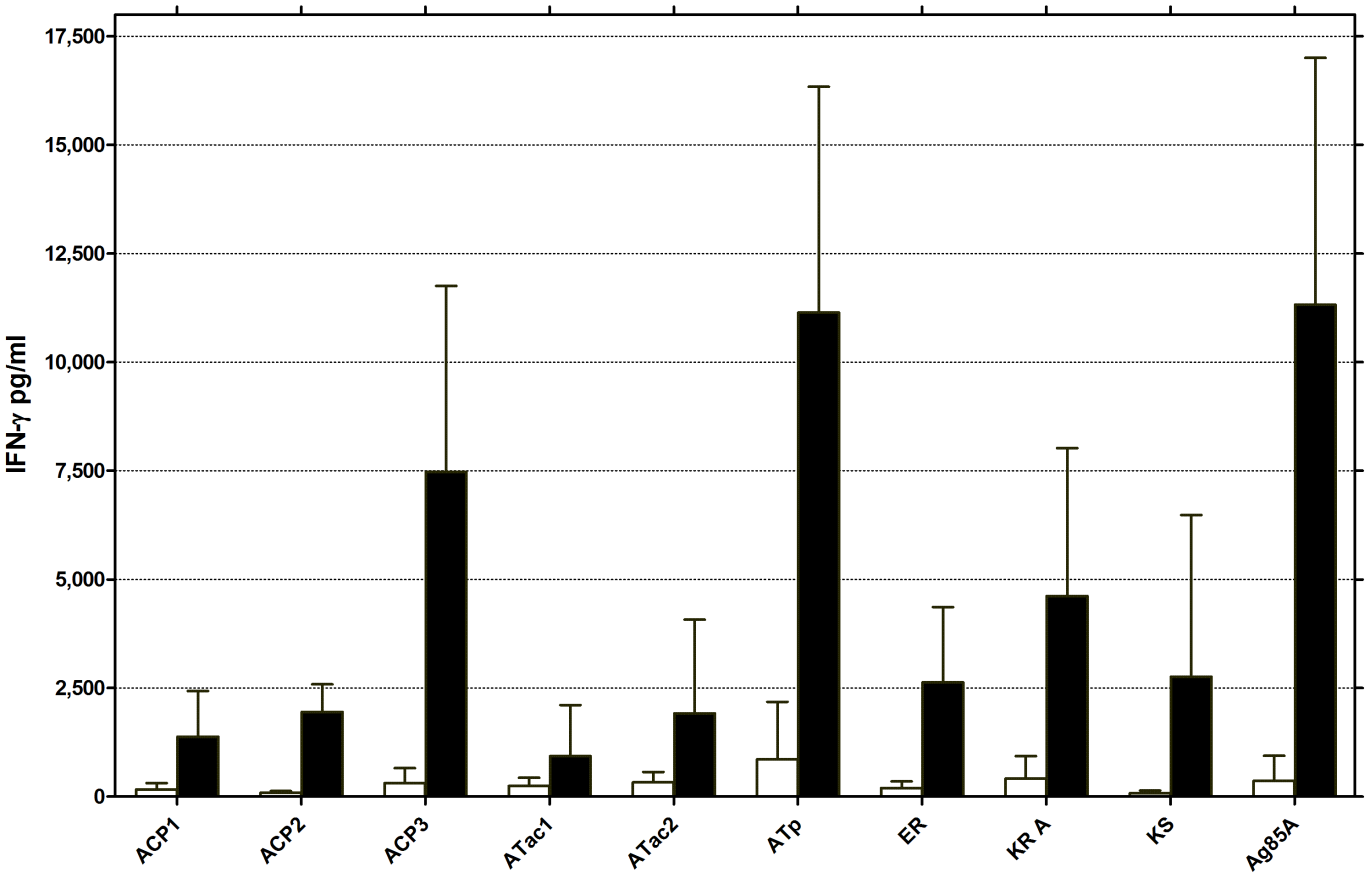
Antigen-specific IFN-γ production in naïve and vaccinated mice, as tested by ELISA. IFN-γ levels in 72 hr spleen cell culture supernatants of C57BL/6 mice vaccinated against the 9 PkS domains using the pDNA prime/protein boost protocol and stimulated *in vitro* with the corresponding recombinant protein antigen (5 µg/ml). Results represent mean ± SD IFN-γvalues (pg/ml) of 4–6 mice tested individually. Data are representative of one of three experiments.

Cytokine levels of the other Th1 cytokine IFN-γ were analyzed in 72 h spleen cell culture supernatants (**Figure 4**). Vaccination against all nine Pks domains induced some antigen-specific IFN-γ responses. Whereas vaccination against ACP1, ACP2, ATac1, ATac2, ER and KS resulted in mean IFN-γ levels of 2.500 pg/ml at most, responses against KR A and ACP3 mounted to 5.000 pg/ml and 7.500 pg/ml respectively. Finally vaccination against ATp and Ag85A resulted in mean IFN-γ levels of more than 10.000 pg/ml.

### Enumeration of antigen-specific IFN-γ producing ELISPOT cells in mice vaccinated against nine Pks domains and MUL4987 using a DNA prime-protein boost protocol

**Figure 5:**
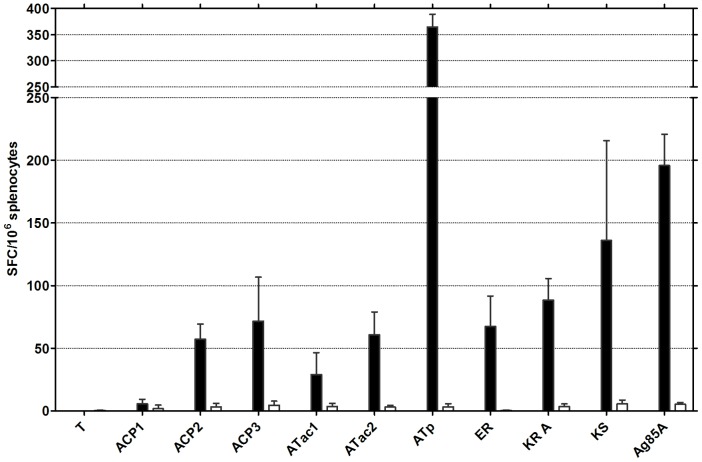
Antigen-specific IFN-γ production in naïve and vaccinated mice, as tested by ELISPOT. Number of IFN-γ producing cells in C57BL/6 mice vaccinated against the 9 PkS domains using the pDNA prime/protein boost protocol and stimulated *in vitro* with the corresponding recombinant protein antigen (5 µg/ml). Results represent mean ± SD of spot forming cells/10^6^ cells of 4–6 mice tested individually as detected in a 48 hr ELISPOT. Data are representative of one of two experiments. White columns: cells without Ag stimulation; black columns: cells stimulated for 48 hr with corresponding antigen.

The number of IFN-γ producing cells was also examined by ELISPOT (**Figure 5**). Some IFN-γ producing cells could be detected after vaccination against all Pks domains, except ACP1. High numbers (between 150 and 200 SFC/10^6^ cells) were measured in response to KS and Ag85A and highest numbers were observed in response to ATp (350 SFC/10^6^ cells).

### Enumeration of AFB in footpad of vaccinated mice, six weeks after *M. ulcerans* challenge

Mice were challenged in the footpad with virulent *M. ulcerans* 1615 six weeks after the protein boost and the number of AFB was enumerated 6 weeks later.

**Figure 6:**
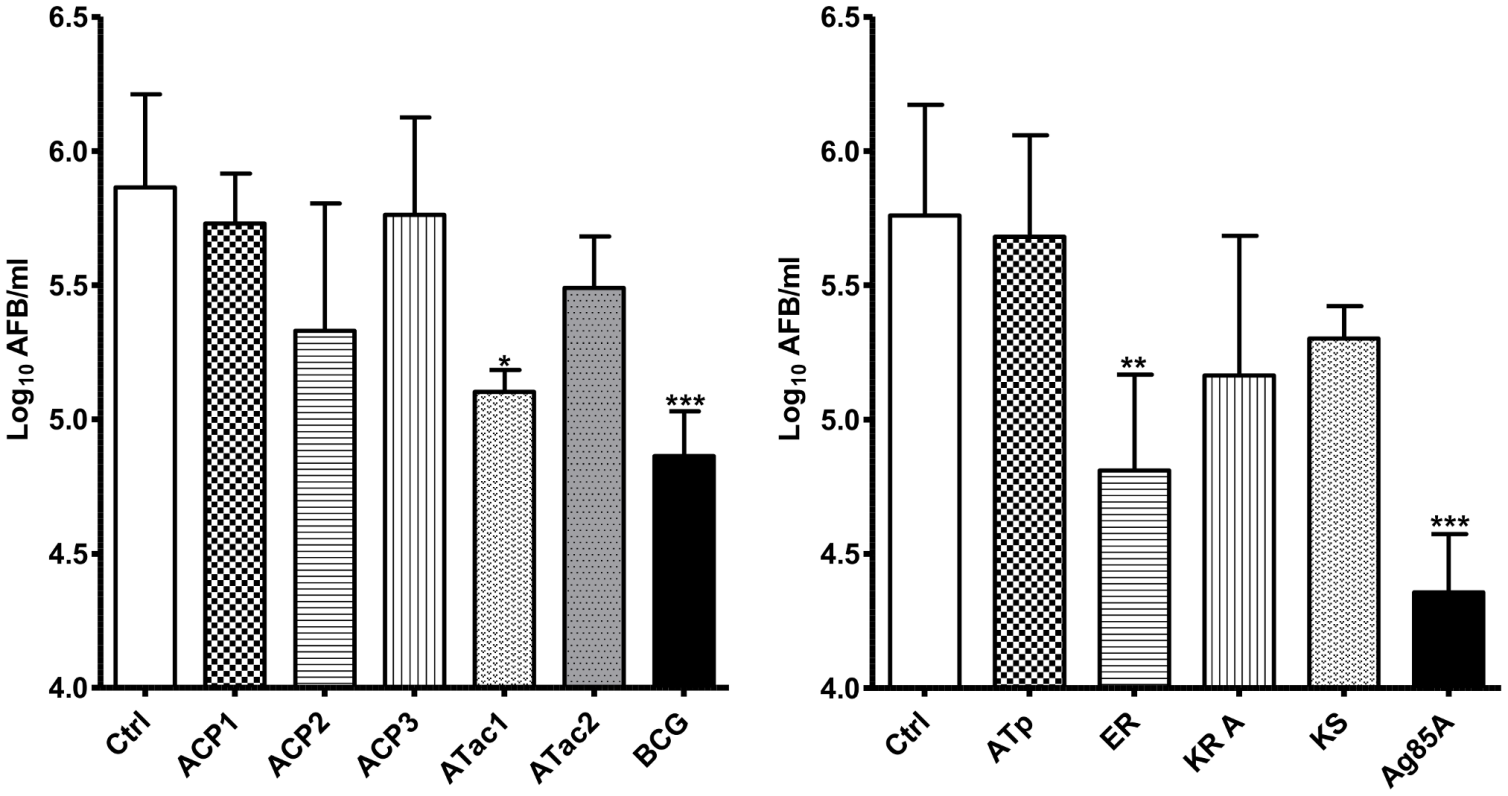
Number of AFB in footpad of naïve or vaccinated mice, six weeks after challenge. Enumeration of acid fast bacteria (AFB) in footpad of naïve or vaccinated mice, challenged 6 weeks after the protein boost with 10^5^ AFB of *M. ulcerans* 1615 and sacrificed another 6 weeks later. Bars represent mean AFB ± SD in footpad of 5 mice tested individually. Numbers were converted to log_10_ AFB/footpad for statistical analysis.

Vaccination against the ER domain, encoding an enoyl reductase, conferred significant protection at this early time point after challenge. Confirming previous findings, vaccination against Ag85A and vaccination with BCG also resulted in significantly reduced AFB numbers in footpad as compared to AFB numbers in naive mice (**Figure 6**).

### Monitoring protection in long term survival experiments

Protection was also evaluated in ten mice/group by monitoring footpad swelling after *M. ulcerans* 1615 infection. The swelling was measured with a calibrated Oditest apparatus and animals were euthanized when footpad swelling exceeded 4 mm according to the rules of the local animal ethics committee.

**Figure 7:**
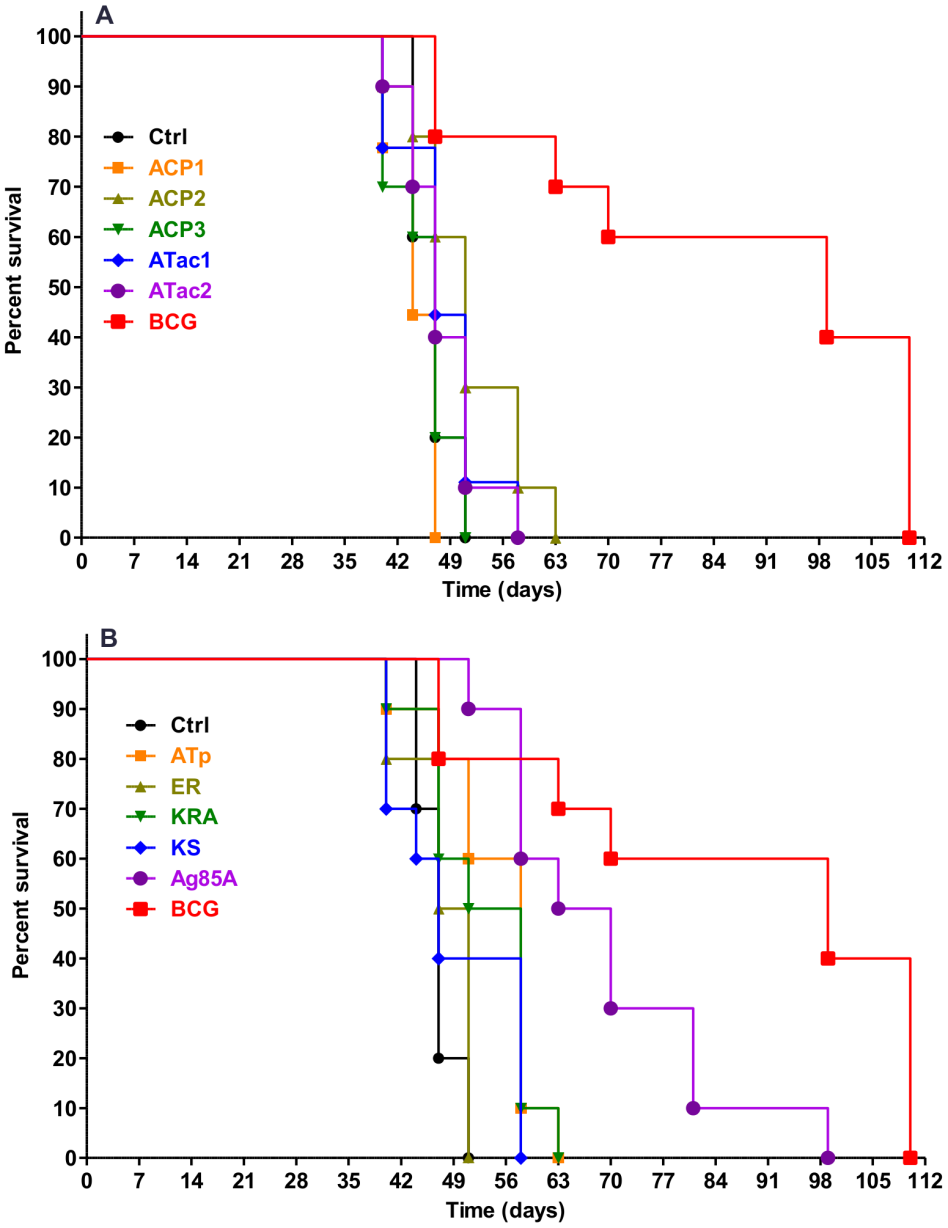
Survival of C57BL/6 mice vaccinated against Pks domains and challenged with *M. ulcerans* 1615. Ten mice/group were challenged 6 weeks after the protein boost with 10^5^ AFB of *M. ulcerans* 1615 and subsequently monitored for 112 days for footpad swelling after *M. ulcerans* 1615 infection. Animals were euthanized when footpad swelling exceeded 4 mm. Figure shows the percentage of surviving mice during the 112 day follow-up. A: Survival curves of unvaccinated (ctrl) mice or mice vaccinated with ACP1, ACP2, ACP3, ATac1, ATac2 or *M. bovis* BCG. B: Survival curves of unvaccinated (ctrl) mice or mice vaccinated with ATp, ER, KR A, KS, Ag85A or *M. bovis* BCG.

Of all the PKS domains, only vaccination against ATp conferred a modest, but significant protection as measured by a delay in footpad swelling and median survival time increased from 47 days in the control group to 58 days in mice that received the ATp vaccine. Vaccination against Ag85A (MST 66 days) and vaccination with BCG (MST 99 days) significantly prolonged the survival time (**Figure 7** **and Table S1**).

## Discussion

Buruli ulcer is a neglected tropical disease [23] for which there is no effective vaccine [2]. The *M. bovis* BCG vaccine, used for the prevention of tuberculosis, has been reported to offer a short-lived protection against the development of skin ulcers [24], [25] and to confer significant protection against disseminated cases of BU, e.g. osteomyelitis, both in children and in adults [26], [27]. Also in mice, BCG vaccine protects to some extent against infection with *M. ulcerans* [15] although a booster vaccination with the same BCG vaccine cannot increase the protective effect and mice finally succumb to the infection [19]. We have previously shown that vaccination with plasmid DNA encoding Ag85A from *M. bovis* BCG can protect, albeit transiently, C57BL/6 mice against footpad challenge with *M. ulcerans* [15]. Antigen 85 is a major secreted component in the culture filtrate of many mycobacteria such as *M. bovis* BCG, *M. tuberculosis* and *M. avium* subsp. *paratuberculosis* [28]. The antigen 85 complex (Ag85) of *M. tuberculosis* is actually a family of three proteins, Ag85A, Ag85B and Ag85C, which are encoded by three distinct but highly paralogous genes and that display an enzymatic mycolyl-transferase activity, involved in cell wall synthesis [29], [30]. Using a DNA prime/protein boost regimen, we have reported that a species specific vaccine composed of Ag85A from *M. ulcerans* was more effective than a vaccine composed of Ag85A of *M. bovis* BCG, conferring a protection, comparable to the protection conferred by the BCG vaccine [18].

Mycolactone is poorly immunogenic, but some of the polyketide synthase domains involved in its synthesis do induce antibodies in BU patients and healthy controls living in endemic regions of Buruli ulcer [31] [14]. Using a plasmid DNA prime/recombinant protein boost protocol, we have confirmed their immunogenicity in an experimental mouse model and have shown that vaccination can induce strong antigen-specific antibodies and Th1 type cytokine responses. Furthermore, a modest protection against a challenge infection with virulent *M. ulcerans* 1615 could be observed in mice vaccinated against ER (reduced AFB numbers early after challenge) and ATp (delayed footpad swelling and increased median survival time). Interestingly, ER was the only *M. ulcerans*-specific antigen leading to an IgG response discriminating ulcerative patients from endemic controls and antibodies against ATp could distinguish healthy controls living in endemic regions of Buruli ulcer from healthy controls living in a non-endemic region [14]. To what extent antibodies directed against these PKS domains can actually inhibit ML synthesis is not clear, but this question certainly warrants further analysis. Two studies have reported that the mycolactone PKS multienzymes are associated with the mycobacterial cell wall [31], [32]. Each mycolactone PKS domain is a component of a large, contiguous polypeptide that makes up the complete multienzyme. The structure of this enzyme is not known, but it is possible that some of the component domains might be orientated such that they are more readily accessed by host immune cells than others.

Based on biopsy specimens, *M. ulcerans* was originally described as an extracellular bacillus. However, the pathogen has an initial intracellular growth phase in macrophages and therefore, recognition of this early intracellular stage by an effective Th1 type immune response, could be an effective means to control the initial infection [33]. Following this proliferation phase within macrophages, *M. ulcerans* induces the lysis of the infected host cells and mycolactone-associated cytotoxicity is responsible for its subsequent extracellular localization [34]–[36]. [37], [38]. This extracellular phase suggests that humoral responses might also be important for protection against *M. ulcerans*. However, as for *M. tuberculosis* infection, the correlates of protection against *M. ulcerans* infection are also unknown. In our study, strongest protection was conferred by vaccines [perhaps use ‘antigens’ instead of ‘vaccines’??] that induced consistently strong cellular and humoral responses (see also Table S2). However, to formally proove the importance of these two cell compartments, transfer studies with specific antibodies or T cell populations would be needed. Finally, even the most immunogenic vaccine can exert its protective effect only when its cognate epitopes are generated and presented to the sensitized immune system upon infection. We speculate that the temporal differences in protection conferred by the ER and ATp vaccine might be related to variations in this antigenic presentation. In favour of this hypothesis is the finding that at the early time point after *M. ulcerans* infection, infected control mice produce stronger IFN-γ responses to the ER than to ATp domain (data not shown).

Hence, a combination vaccine targeting both the early intracellular and the subsequent extracellular stage, through the induction of strong Th1 T cells and antibodies respectively, may be needed to control the infection effectively. A vaccine composed of the mycolyl transferase Ag85A combined with the most immunogenic polyketide synthase domains is an interesting possibility that requires further study. Also priming with pDNA followed by boosting with *M. bovis* BCG or live, attenuated mycolactone-negative *M. ulcerans* mutants [33] could be envisaged, as has been reported in tuberculosis vaccine development [39], [40].

## Supporting Information

Figure S1 **Antigen-specific IgG antibodies in naïve and vaccinated mice.** IgG antibodies in C57BL/6 mice vaccinated twice with pDNA encoding the nine Pks synthase domains and boosted with the homologous recombinant protein. Sera were collected six weeks after the protein boost and tested by ELISA, using serial twofold dilutions, starting at 1∶50. Open circles: naive mice, closed circles vaccinated mice. Results presented as mean O.D. values ± SD of 4–6 mice tested individually.
(TIF)

Table S1 **Median survival time of C57BL/6 mice vaccinated against Pks domains and challenged with virulent** ***M. ulcerans*** **.**
(DOCX)

Table S2 **Summary of immunogenicity and protective efficacy of DNA vaccines encoding nine Pks domains or Ag85A.**
(DOCX)
