# Supplementary material for: Analysis of the Vaccine Potential of Plasmid DNA Encoding Nine Mycolactone Polyketide Synthase Domains in Mycobacterium ulcerans Infected Mice
Source: PLoS Negl Trop Dis. 2014 Jan 2;8(1):e2604. doi: 10.1371/journal.pntd.0002604 (PMC3879250; doi:10.1371/journal.pntd.0002604)
Supplement: Table S1 — Median survival time of C57BL/6 mice vaccinated against Pks domains and challenged with virulent M. ulcerans . (DOCX) [file pntd.0002604.s002.docx]

**Table S1**

**Median survival time of C57BL/6 mice vaccinated against Pks domains and challenged with virulent *M. ulcerans***

| **Antigen used for vaccination** | **Median Survival time** | **Ratio to control** | **p**  **(Mantel-Cox)** | **significance** |
| --- | --- | --- | --- | --- |
| **Control** | **47** |  |  |  |
| **ACP1** | **44** | **1.068** | **0.159** | **ns** |
| **ACP2** | **51** | **0.922** | **0.055** | **ns** |
| **ACP3** | **47** | **1** | **0.771** | **ns** |
| **ATac1** | **47** | **1** | **0.268** | **ns** |
| **ATac2** | **47** | **1** | **0.353** | **ns** |
| **ATp** | **58** | **0.810** | **0.003** | ****** |
| **ER** | **49** | **0.959** | **0.272** | **ns** |
| **KR A** | **54.5** | **0.862** | **0.017** | ***** |
| **KS** | **47** | **1** | **0.296** | **ns** |
| **Ag85A** | **66.5** | **0.707** | **< 0.0001** | ******* |
| ***M. bovis* BCG** | **99** | **0.475** | **0.0002** | ******* |

*: p < 0.05 **: p < 0.01 ***: p < 0.001 ns: statistically not significant, as compared to MST of control mice
