# Supplementary material for: Analysis of the Vaccine Potential of Plasmid DNA Encoding Nine Mycolactone Polyketide Synthase Domains in Mycobacterium ulcerans Infected Mice
Source: PLoS Negl Trop Dis. 2014 Jan 2;8(1):e2604. doi: 10.1371/journal.pntd.0002604 (PMC3879250; doi:10.1371/journal.pntd.0002604)
Supplement: Table S2 — Summary of immunogenicity and protective efficacy of DNA vaccines encoding nine Pks domains or Ag85A. (DOCX) [file pntd.0002604.s003.docx]

**Table S2.**

**Summary of immunogenicity and protective efficacy of DNA vaccines encoding nine Pks domains or Ag85A.**

| **Domain** | **IL-2** | **IFN-γ** | **IgG** | **Protection** |
| --- | --- | --- | --- | --- |
| ACP1 | + | + | - | - |
| ACP2 | + | + | - | - |
| ACP3 | + | ++ | - | - |
| ATac1 | + | + | - | - |
| ATac2 | +++ | + | + | - |
| ATp | +++ | +++ | ++ | + (late) |
| ER | + | + | + | + (early) |
| KR A | ++ | ++ | - | - |
| KS | ++ | + | - | - |
| Ag85A | +++ | +++ | ++ | ++ (early and late) |
